# Endocannabinoid Mediates Excitatory Synaptic Function of β-Neurexins. Commentary: β-Neurexins Control Neural Circuits by Regulating Synaptic Endocannabinoid Signaling

**DOI:** 10.3389/fnins.2016.00203

**Published:** 2016-05-20

**Authors:** Hansen Wang

**Affiliations:** Faculty of Medicine, University of TorontoToronto, ON, Canada

**Keywords:** β-neurexins, endocannabinoid, synaptic plasticity, neural circuits, LTP, autism, Alzheimer disease, fragile X syndrome

## Introduction

Synaptic cell-adhesion molecules and their interactions with other molecular pathways affect both synapse formation and its function (Varoqueaux et al., 2006; Sudhof, 2008; Bemben et al., 2015a). Neurexins are presynaptic cell-adhesion molecules that interact with neuroligins and other postsynaptic partners. Neurexins are encoded by three genes, each of which encodes a long and short isoform, termed α- and β-neurexins, respectively (Sudhof, 2008). Interestingly, despite studies linking neurexins to autism and other neuropsychiatric disorders (Leone et al., 2010; Rabaneda et al., 2014), the precise cellular mechanisms underlying the role of neurexins in cognition remain poorly understood.

**Figure 1:**
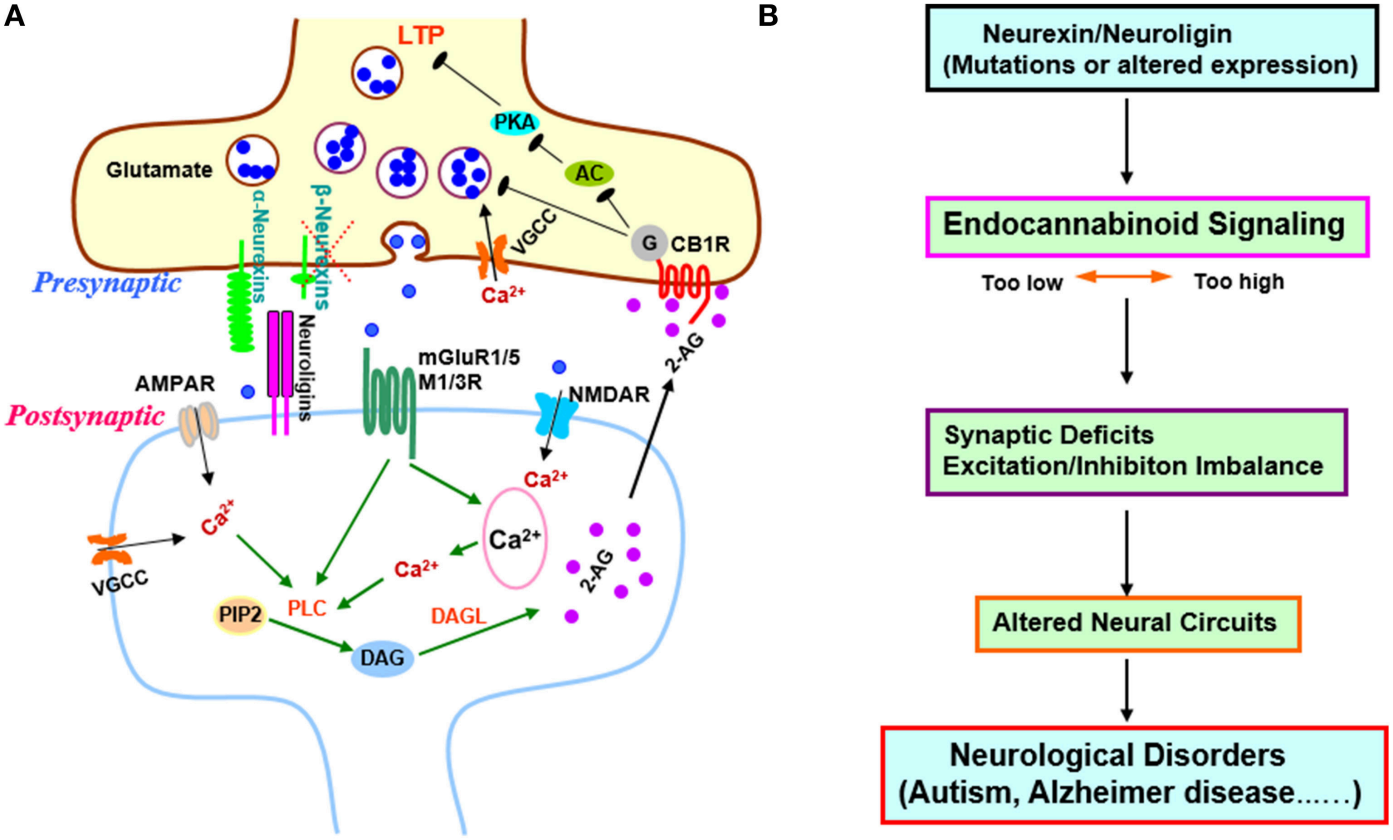
**Transsynaptic regulation of endocannabinoid signaling by β-neurexins and its implications in synaptic plasticity and diseases. (A)** Regulation of excitatory synaptic strength by β-neurexins via endocannabinoid system. Anderson et al. demonstrated that presynaptic β-neurexins regulate endocannabinoid signaling by controlling postsynaptic endocannabinoid 2-AG synthesis. When β –neurexins are removed, 2-AG synthesis is disinhibited, presynaptic CB1Rs are activated, and synaptic strength is decreased (Anderson et al., 2015). In addition, the AC-PKA dependent LTP in burst-firing neurons is blocked, which may account for the impaired contextual memory in hippocampal CA1 β-neurexin knockout mice. β-neurexins act as a brake on endocannabinoid signaling possibly via transsynaptic interaction with postsynaptic neuroligin isoforms that exclusively bind to β-neurexins, but not a-neurexins (Anderson et al., 2015). β-neurexins might downregulate tonic endocannabinoid signaling through mGluR1/5 or M1/M3 receptors since activation of those GPCRs is known to trigger 2-AG production via PLC pathway (Varma et al., 2001; Chevaleyre et al., 2006; Heifets and Castillo, 2009; Kano et al., 2009; Castillo et al., 2012; Rinaldo and Hansel, 2013; Martin et al., 2015). This regulation might also involve VGCCs, NMDARs, or AMPARs as Ca^2+^ influx through these channels could facilitate PLC-DAGL mediated 2-AG production (Ohno-Shosaku et al., 2005; Castillo et al., 2012). The exact postsynaptic partners of β-neurexins in this process await to be identified. **(B)** The regulation of endocannabinoid signaling by β-neurexins supports neurexins/neuroligins-endocannabinoid signaling as a common pathomechanism in cognitive disorders (Krueger and Brose, 2013; Anderson et al., 2015). Abnormalities in this signaling pathway could disrupt synapses and neural circuits, and contribute to neurological and psychiatric diseases (Chubykin et al., 2005; Tabuchi et al., 2007; Katona and Freund, 2008; Sudhof, 2008; Gogolla et al., 2009; Bot et al., 2011; Etherton et al., 2011; Foldy et al., 2013; Singh and Eroglu, 2013; Rothwell et al., 2014; Sindi et al., 2014; Aoto et al., 2015; Bedse et al., 2015; Born et al., 2015; Di Marzo et al., 2015; Parsons and Hurd, 2015; Wang and Doering, 2015; Wang et al., 2015; Bemben et al., 2015b; Chanda et al., 2016). Abbreviations: 2-AG, 2-arachidonoyl-sn-glycerol; AC, adenylyl cyclase; AMPAR, a-amino-3-hydroxy-5-methyl-4-isoxazolepropionic acid receptor; CB1R, cannabinoid receptor 1; DAG, diacylglycerol; DAGL, diacylglycerol lipase; LTP, long-term potentiation; M1/3R, muscarinic acetylcholine receptor 1/3; mGluR, metabotropic glutamate receptor; NMDAR, N-methyl-D-aspartate receptor; PIP2, phosphatidylinositol 4, 5-bisphosphate; PKA, protein kinase A; PLC, phospholipase C; VGCC, voltage-gated Ca^2+^ channels.

Since most biochemical studies of neurexins have focused on β-neurexins, investigating the synaptic actions of β-neurexins is particularly imperative. In their timely *Cell* article, Anderson et al. reported that β-neurexins selectively modulate synaptic strength at excitatory synapses by regulating postsynaptic endocannabinoid synthesis, describing an unexpected trans-synaptic mechanism for β-neurexins to control neural circuits via endocannabinoid signaling (Anderson et al., 2015; Summarized in Figure 1A).

## β-Neurexins regulate excitatory neurotransmission via endocannabinoid signaling

Functional study of neurexins represents a major technical challenge due to their diversity and complexity. To study the specific role of β-neurexins, Anderson et al. generated conditional knockout mice of all β-neurexin genes. Using electrophysiological and pharmacological approaches, the authors elegantly analyzed neurotransmission and synaptic strength in preparations of cultured cortical neurons and acute subiculum slices from those β-neurexin knockout mice (Anderson et al., 2015).

Can β-neurexins be specifically involved in excitatory or inhibitory neurotransmission? In cultured cortical neurons, Anderson et al. found that β-neurexin knockout decreased the excitatory synapse parameters including AMPA receptor- and NMDA receptor-mediated excitatory postsynaptic currents (EPSCs), release probability and action-potential induced calcium influx, but had no effect on GABA receptor-mediated inhibitory postsynaptic currents (IPSCs; Anderson et al., 2015). Consistently, β-neurexin knockout decreased spontaneous miniature EPSCs (mEPSCs) and lowered the surface GluA1 AMPARs, but had no effect on miniature IPSCs (mIPSCs) (Anderson et al., 2015). These data indicate that β-neurexins are selectively essential for neurotransmission at excitatory synapses. Importantly, the impaired mEPSCs could be rescued by re-expression of neurexin-1β, but not by increased expression of neurexin-1α, suggesting that modulation of excitatory neurotransmission by β-neurexins, despite their lower abundance, is independent of α-neurexins (Anderson et al., 2015).

How can β-neurexins modulate excitatory transmission? As their previous study has suggested that neuroligin-3 is specifically required for tonic endocannabinoid signaling at inhibitory synapses (Foldy et al., 2013), Anderson et al. hypothesized that β-neurexins, the presynaptic interactor of neuroligin-3, might regulate neurotransmission via endocannabinoid system. To test this, the authors pharmacologically manipulated the endocannabinoid system in cultured cortical neurons. Indeed, treatment with a cannabinoid receptor 1 (CB1R) antagonist, enhanced the mEPSC frequency in β-neurexin knockout neurons, but had no effect in control neurons; the CB1R agonist caused less decrease in mEPSC frequency in β-neurexin knockout neurons than in control ones (Anderson et al., 2015). These findings indicate that β-neurexin knockout enhances basal endocannabinoid tone and tonic presynaptic CB1R activation, further revealing a link of the neurexins/neuroligins complex to endocannabinoid signaling. As presynaptic CB1R activation are known to inhibit presynaptic Ca^2+^ channels and decrease neurotransmitter release (Castillo et al., 2012), the authors conclude that β-neurexins might control excitatory neurotransmission through downregulating endocannabinoid system and the impaired excitatory neurotransmitter release is at least partially due to enhanced endocannabinoid signaling in absence of β-neurexins (Anderson et al., 2015).

How does β-neurexin knockout increase tonic endocannabinoid signaling at excitatory synapses? The examination of CB1R levels detected no changes in β-neurexin knockout neurons (Anderson et al., 2015), suggesting that β-neurexin knockout may affect endocannabinoid synthesis. To identify which of the two major endocannabinoids—2-arachidonoylglycerol (2-AG) and anandamide—is affected by β-neurexin knockout, Anderson et al. compared the effects of bath application of each endocannabinoid, and found that the enhanced endocannabinoid tone might be caused by the increase of 2-AG as exogenous 2-AG produced little additional inhibition on mEPSCs in β-neurexin knockout neurons. 2-AG is synthesized via a postsynaptic phospholipase C-dependent pathway (Anderson et al., 2015). Unsurprisingly, inhibition of 2-AG synthesis in postsynaptic neurons with phospholipase C inhibitor rescued mEPSC frequency and restored the sensitivity of CB1Rs to exogenous 2-AG in β-neurexin knockout neurons, further confirming that loss of β-neurexins cause synaptic phenotypes via presynaptic CB1R activation by elevated postsynaptic 2-AG production (Anderson et al., 2015). Notably, the postsynaptic partners of β-neurexins in regulating endocannabinoid synthesis remain unknown (Figure 1A).

Impressively, in acute subiculum slices, Anderson et al. found that presynaptic β-neurexin knockout in CA1 pyramidal neurons selectively decreases excitatory synaptic strength at burst-firing subiculum neurons, at least in part, by enhancing tonic endocannabinoid signaling, indicating that β-neurexins also control endocannabinoid system *in vivo* (Anderson et al., 2015). Particularly, β-neurexin knockout selectively blocked long-term potentiation (LTP) in burst-firing neurons (Anderson et al., 2015). LTP is induced by presynaptic activation of PKA in burst-firing neurons of the subiculum (Wozny et al., 2008). Activation of CB1Rs, which are Gi/o protein-coupled receptors, inhibits adenylyl cyclases/PKA (Castillo et al., 2012) and possibly blocks presynaptic LTP. The authors next demonstrated both CB1R antagonist and 2-AG synthesis inhibitor rescued the LTP impairment caused by β-neurexin knockout, firstly linking endocannabinoid signaling to presynaptic LTP of excitatory synapses (Anderson et al., 2015; Figure 1A). Further research is needed to investigate the mechanism underlying the cell-specific function of β-neurexins in burst-firing neurons relative to regular-firing ones.

Finally, the authors showed that deleting β-neurexins from the hippocampal CA1 region selectively impaired mouse contextual fear memory, indicating that β-neurexins in hippocampal CA1 neurons is important for learning and memory (Anderson et al., 2015). However, the behavioral evidence is still limited. Additionally, the authors did not confirm the involvement of endocannabinoid system in behavioral deficits caused by hippocampal β-neurexin knockout.

Altogether, Anderson et al. exquisitely revealed that β-neurexins have a unique role in transynaptic modulation of endocannabinoid tone at excitatory synapses, which is essential for synaptic plasticity and behaviors, thus mechanistically linking β-neurexins to cognitive function (Anderson et al., 2015).

## Future perspective

Investigating the synaptic function of neurexins/neuroligins is crucial to elucidate the pathomechanisms of diseases associated with these cell-adhesion molecules. The discovery of transynaptic modulation of endocannabinoid signaling by β-neurexins, not only provides insights into the molecular mechanisms underlying neural circuits, but also helps understand synaptopathies in cognitive diseases.

Endocannabinoid system regulates neural circuits and offers therapeutic opportunities for neuropsychiatric diseases (Castillo et al., 2012; Wyrofsky et al., 2015). The neurexins/neuroligins-endocannabinoid signaling pathway likely modulates circuit dynamics in distinct brain regions and may implicate many brain disorders (Figure 1B). The conditional knockout mice combined with other genetic or pharmacological approaches will provide useful tools for investigating this pathway in neural circuits and its behavioral and therapeutic relevance. Much more work will be required, but the study highlighted herein is encouraging in this direction.

## Author contributions

The author confirms being the sole contributor of this work and approved it for publication.

### Conflict of interest statement

The author declares that the research was conducted in the absence of any commercial or financial relationships that could be construed as a potential conflict of interest.
